# Whole genome sequence analysis of the TALLYHO/Jng mouse

**DOI:** 10.1186/s12864-016-3245-6

**Published:** 2016-11-11

**Authors:** James Denvir, Goran Boskovic, Jun Fan, Donald A. Primerano, Jacaline K. Parkman, Jung Han Kim

**Affiliations:** Department of Biomedical Sciences, Joan C. Edwards School of Medicine, Marshall University, 1700 3rd Ave. #435K BBSC, Huntington, WV 25755 USA

**Keywords:** Whole genome sequencing, Obesity, Type 2 diabetes, Mouse model, TALLYHO, Quantitative Trait Loci

## Abstract

**Background:**

The TALLYHO/Jng (TH) mouse is a polygenic model for obesity and type 2 diabetes first described in the literature in 2001. The origin of the TH strain is an outbred colony of the Theiler Original strain and mice derived from this source were selectively bred for male hyperglycemia establishing an inbred strain at The Jackson Laboratory. TH mice manifest many of the disease phenotypes observed in human obesity and type 2 diabetes.

**Results:**

We sequenced the whole genome of TH mice maintained at Marshall University to a depth of approximately 64.8X coverage using data from three next generation sequencing runs. Genome-wide, we found approximately 4.31 million homozygous single nucleotide polymorphisms (SNPs) and 1.10 million homozygous small insertions and deletions (indels) of which 98,899 SNPs and 163,720 indels were unique to the TH strain compared to 28 previously sequenced inbred mouse strains. In order to identify potentially clinically-relevant genes, we intersected our list of SNP and indel variants with human orthologous genes in which variants were associated in GWAS studies with obesity, diabetes, and metabolic syndrome, and with genes previously shown to confer a monogenic obesity phenotype in humans, and found several candidate variants that could be functionally tested using TH mice. Further, we filtered our list of variants to those occurring in an obesity quantitative trait locus, *tabw2,* identified in TH mice and found a missense polymorphism in the *Cidec* gene and characterized this variant’s effect on protein function.

**Conclusions:**

We generated a complete catalog of variants in TH mice using the data from whole genome sequencing. Our findings will facilitate the identification of causal variants that underlie metabolic diseases in TH mice and will enable identification of candidate susceptibility genes for complex human obesity and type 2 diabetes.

**Electronic supplementary material:**

The online version of this article (doi:10.1186/s12864-016-3245-6) contains supplementary material, which is available to authorized users.

## Background

The high prevalence of obesity and type 2 diabetes is a serious public health issue that is associated with devastating health consequences such as cardiovascular diseases [1, 2]. The World Health Organization estimated that more than 10 % of the world’s adult population (200 million men and 300 million women) was obese (body mass index ≥30 kg/m^2^) in 2008 (http://www.who.int/mediacentre/factsheets/fs311/en/) and that, in 2012, 347 million people had diabetes, 90 % of whom had type 2 diabetes (http://www.who.int/mediacentre/factsheet/fs312/en/). Genetic predisposition is recognized as a major risk factor for the development of obesity and type 2 diabetes; estimates of the heritability range from 50 % to 60 % for both diseases [3]. Therefore, identification of underlying susceptibility genes would define potential targets and pathways for intervention and treatment of obesity and type 2 diabetes.

The genetics of human obesity and type 2 diabetes is complex [4, 5]. It involves multiple susceptibility genes and their interactions with environmental factors [6]. Animal models that share both physiologic and genetic similarity with humans are used in obesity and type 2 diabetes research to minimize the confounding effects of heritability, genetic heterogeneity and environment that determine these diseases in humans [7, 8].

The TALLYHO/Jng (TH) mouse is a polygenic inbred model for human obesity and type 2 diabetes [9]. TH mice manifest many of the disease phenotypes observed in human obesity and type 2 diabetes including hyperleptinemia, hyperinsulinemia, insulin resistance, glucose intolerance, hyperlipidemia, and hyperglycemia [10]. These mice also exhibit increased islet insulin secretion in response to glucose and β-cell mass [11]. The genetic basis for obesity and type 2 diabetes in TH mice has been studied using outcross experiments with normal strains which has led to the identification of multiple quantitative trait loci (QTLs) linked to adiposity and hyperglycemia [10].

In addition to genetic analysis, the TH mouse has been used in the development of therapeutic agents for obesity and type 2 diabetes [12, 13] and has also served as a model system for many diabetes and obesity related abnormalities, including decreased exercise capacity [14], impaired wound healing [15–17], periodontitis [18], tissue susceptibility to hypoxia [19, 20], bone loss [21, 22], circadian disruption [23], and vasculature abnormalities [24–26].

In this study, we sequenced the whole genome of the TH mouse using next-generation sequencing with the goal of identifying genome-wide sets of single nucleotide polymorphisms (SNPs) and small insertions and deletions (indels), a subset of which are present exclusively in the TH mouse (private variants). A complete catalogue of TH genomic variants (public and private) will aid the identification of causative variants and genes underlying the diseases observed in TH mice, which will maximize the relevancy of this model for human obesity and type 2 diabetes. Knowing the distribution of variants across the genome of the TH mouse vs. other classic and wild-derived inbred strains [27] may identify naturally occurring gene variant sets that can represent model genes for the complex human obesity and type 2 diabetes. As a proof of principle, we integrated our genome variant data with genetic mapping data and identified a variant in cell death-inducing DFFA-like effector c (*Cidec*) whose effect on protein function we characterized.

## Methods

### Description and origin of the TH mouse strain

The TH mouse is originally derived from an outbred colony of the Theiler Original strain in which two male mice spontaneously became polyuric, glucosuric, hyperinsulinemic, and hyperglycemic (Harrow, United Kingdom) [28]. In 1994, male diabetic progeny and apparently normal female mice were imported into The Jackson Laboratory (TJL) (Bar Harbor, ME, USA) and an inbred strain was then established by selecting for male hyperglycemia by Dr. Jürgen Naggert’s research group at TJL. In 2001, a sub-colony was initiated in our laboratory with breeding pairs from Dr. Naggert’s research colony [10] and used in this study. After arrival at our laboratory, we interbred siblings for 18 generations followed by one generation backcrossing and an additional 14 generations of sibling interbreeding. Since the generation prior to arrival at our laboratory remains unknown, we refer to the generation used for whole genome sequencing in this study as F? + F18N1F14. All animal studies were carried out with the approval of Marshall University Animal Care and Use Committee.

### DNA sequencing, read alignment, and bioinformatics

High quality genomic DNA was extracted from the liver of a male TH mouse from our colony using a Qiagen (Valencia, CA) Genomic-tip 100/G kit. Genomic DNA (1 μg) was sheared using a Covaris (Woburn, MA) S2 instrument to ~350 bp and used to construct a sequencing library using an Illumina (San Diego, CA) DNA Sample Preparation kit according to the manufacturer’s recommended protocol. The library was quantified using ThermoFisher (Waltham, MA) Qubit fluorimetry and sized on an Agilent (Santa Clara, CA) Bioanalyzer DNA chip. The resulting whole genome library (8 pmole) was amplified on a flow cell using an Illumina cBot cluster station and then sequenced on an Illumina HiSeq 1000 in the Marshall University Genomics Core Facility. Three sequencing runs (one 2x50 bp paired-end and two 2x100 bp paired-end) were performed in order to obtain adequate depth of coverage.

Sequencing reads were aligned to the C57BL/6J (B6) reference genome (GRCm38, mm10) using Bowtie2 v2.1.0 [29]. Our variant calling pipeline was based on that developed by Wong et al. [30] to call variants in the FVB/NJ mouse strain. Briefly, duplicate reads were removed with SAMtools v0.1.18 [31] using the “samtools rmdup” command. To improve quality of the variant calling, local realignment around insertions and deletions was performed using GATK v3.2.2 [32] by first running the “RealignerTargetCreator” command and then the “IndelRealigner” command. SNPs and indels were called by generating a pileup using the “samtools pileup” command with options uEDS, and piping the results to the “bcftools view” command with options “-p 0.99 –vbcgN”. Variants were filtered using the VCFtools package [33] version 0.1.12. In order to maintain maximal consistency with the Mouse Genome Project (MGP) [34], we used the same options as in Wong et al. [30].

We identified variants in the TH strain that did not occur in any of the 28 mouse strains published in the MGP (Additional file 1: Table S1), which we term “private” variants, following Keane et al. [34]. To do this, we generated a list of all genomic locations of TH variants in the form of a bed file, and then performed variant calling on the bam files for the 28 MGP mouse strains at those locations, using SAMTools’ “–l” option along with the same parameters as were used to call TH variants. TH SNPs qualified as private if no MGP strain had the same SNP at the same location, where at least 21 of the 28 strains had a call quality at least 20 and read depth at least 5 at that location. TH indels qualified as private if no MGP strain had a variant at the same genomic location, with the same criteria for call quality and read depth being applied.

We then added functional consequence annotation, including “Sorting Intolerant From Tolerant” (SIFT) [35] prediction of protein function changes of coding variants, using a local installation of Ensembl Variant Effect Predictor (VEP) version 77 [36]. Output from VEP includes classification of each variant using a set of one or more Sequence Ontology (SO) terms [37]. Variants are classified multiple times if they lie within a region intersecting multiple known transcripts. We tabulated the number of variants according to their collection of SO terms, collecting into a single “Multiple Classification Sets” group all variants that had multiple, distinct sets of SO terms due to their presence in multiple transcripts. Since this latter group is large, and may include potentially interesting variants, we identified a set of ten SO terms we call “potentially pathogenic”: *frameshift_variant, inframe_deletion, inframe_insertion, missense_variant, stop_gained, stop_lost, initiator_codon_variant, splice_region_variant, splice_acceptor_variant,* and *splice_donor_variant*. We tabulated the number of variants in the “multiple classification sets” that classified with each of these SO terms for one or more transcripts. The same classification was performed on the private variants. For missense variants, we additionally used Protein Variation Effect Analyzer v 1.1 (PROVEAN) [38] to provide further prediction of the functional effects of protein changes.

In order to uniquely associate a representative SO term with each variant, we chose a single representative SO term for each distinct set of SO terms identified for a given variant in a given transcript, as shown in Additional file 2: Table S2 and Additional file 3: Table S3. For example, 32 SNPs were classified with the two SO terms “intron_variant” and “splice_region_variant”: for these SNPs we chose “splice_region_variant” as the representative term. Using this process, variants occurring in multiple transcripts that had multiple, distinct sets of SO terms associated with them, resulted in having multiple representative SO terms. For such variants, we chose the “most pathogenic” representative SO term, ordering them in the following priority: “stop_gained”, “stop_lost”, “frameshift_variant”, “missense_variant”, “inframe_insertion”, “inframe_deletion”, “splice_acceptor_variant”, “splice_donor_variant”, “splice_region_variant”, “initiator_codon_variant”, “synonymous_variant”, “5_prime_UTR_variant”, “3_prime_UTR_variant”, “mature_miRNA_variant”, “upstream_gene_variant”, “downstream_gene_variant”, “intron_variant”, “non_coding_transcript_variant”, “intergenic_variant”.

In order to compare our variant sets to known associations between genetic variants and human traits for which TH is a model, we first retrieved the Genome Wide Association Study (GWAS) catalog [39] from the European Bioinformatics Institute (EBI) (https://www.ebi.ac.uk/gwas/), and filtered the catalog by the column “DISEASE/TRAIT” for each of the terms “obesity”, “diabetes”, and “metabolic”. For each filtered catalog, we extracted all human Entrez GeneIDs listed in the columns MAPPED GENE(S), UPSTREAM_GENE_ID, and DOWNSTREAM_GENE_ID. Using the Ensembl interface to BioMart [40], we converted these Entrez GeneIDs to Ensembl human gene IDs. Additionally, we retrieved a list of genes from Pigeyre et al. [41] in which mutations have been previously shown to cause a monogenic obesity phenotype in humans, and found Ensembl IDs for these genes using the same tool. For all these human Ensembl gene IDs, we found Ensembl mouse gene IDs of orthologous genes, again using the Ensembl interface to Biomart. We then filtered our variant sets (SNPs, indels, private SNPs, and private indels) to identify potentially pathogenic variants associated with each of these orthologous gene sets.

Aligned reads were uploaded to the Sequence Read Archive (SRA) at the National Center for Biotechnology Information (NCBI) and can be accessed via accession number SRP067703.

### Cell culture, plasmids, transfections, and microscopy

COS-1 cells (ATCC, Manassas, VA) were grown in Dulbecco’s modified Eagle’s medium with L-glutamine, 4.5 g/L glucose and sodium pyruvate (Mediatech, Manassas, VA) supplemented with 10 % (v/v) bovine serum (Sigma, St. Louis, MO) and 100 units/ml penicillin and 100 mg/ml streptomycin (Mediatech, Manassas, VA).

The murine *Cidec* cDNA was procured from GenScript (Piscataway, NJ). A Green Fluorescent Protein fusion, pAcGFP1-CIDEC was created by cloning the *Cidec* cDNA with 5′ HindIII and 3′ BamHI sites upstream and in-frame with the AcGFP1 sequence in pAcGFP1-N1 (Clontech, Mountain View, CA). The entire *Cidec* coding region was included except for the stop codon [42]. Site-directed mutagenesis was used to introduce the missense polymorphism R46S (GeneScript, Piscataway, NJ). Plasmids, pAcGFP1-CIDEC (R46) and pAcGFP1-CIDEC (S46), were transfected into COS-1 cells cultured on 4-well chamber slides (1 x 10^5^ cells per chamber) (Thermo Scientific, Waltham, MA) using Lipofectamine LTX&PLUS (Life Technologies, Grand Island, NY) following the manufacturer’s instruction [43]. COS cells do not express endogenous CIDEC [44]. After transfection, the cells were cultured for 48 h with and without 400 μM BSA-complexed oleic acid (Sigma) [45]. Cells were then washed with PBS, fixed by 4 % paraformaldehyde for 30 min, and permeabilized in 0.05 % (w/v) saponin (Sigma) in PBS for 20 min. Lipids were stained with 1 μg/ml Nile red (Sigma) which partitions with neutral lipids; cells were washed with PBS (6x). Nuclei were labeled by placing coverslips onto slides with Prolong® Gold Antifade Mountant with DAPI (Life Technologies). Coverslips were dried and cells viewed on a Leica SP5 confocal microscope located in the Marshall University Imaging Center. Each experiment was performed in quadruplicate samples. Association of full length *Cidec* with lipid droplets in COS-1 cells was assessed using ImageJ software by performing a co-localization analysis of GFP florescence with Nile red labeled lipid droplets in each z-section of a transfected cell [43]; these results were then averaged to calculate the overall co-localization in a given cell (the ratio of Nile red signal specifically associated with GFP signal to GFP signal). The data were from 12 cell clusters in each condition from two independent experiments. Quantitative data were presented as means ± SEM. Two-tailed Student’s t-tests were performed on data. Differences of *P* < 0.05 were considered significant.

## Results

### Identification of SNPs and indels

**Fig. 1:**
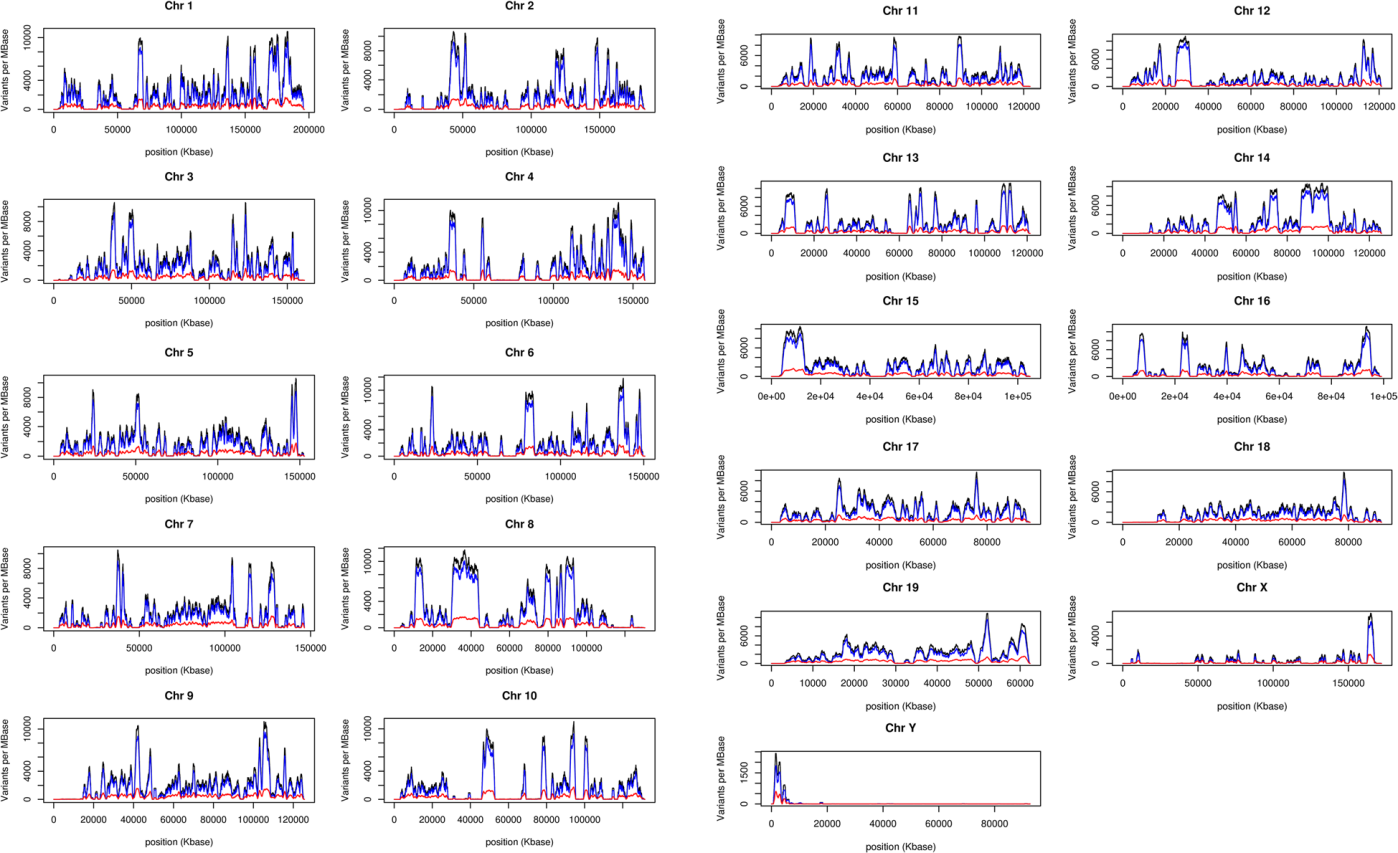
Distribution of detected variants on the TH genome. Total number of variants (black), number of SNPs (blue), and number of indels (red) per megabase as a function of genomic position. These were calculated by sliding a one-megabase window across each chromosome in steps of one kilobase and totaling the number of variants in the window

The whole genome of the TH mouse was sequenced to an average depth of ~64.8X coverage using data from three Illumina paired-end read sequencing runs. The sequencing reads were mapped to the B6 mouse reference genome (GRCm38/mm10). Using a SAMTools-based pipeline [31], we identified 4,370,213 SNPs (4,310,548 of which were homozygous) and 1,213,617 indels (1,065,090 of which were homozygous) genome-wide, relative to the reference strain C57BL/6 (B6). The positional distribution of variants on each chromosome is shown in Fig. 1. To assess the reliability of our data, PCR amplification and Sanger sequencing were applied to 14 homozygous SNPs to determine whether they agreed with next generation sequencing results (Additional file 4: Table S4). We found that all 14 SNPs were consistent with the Illumina sequencing data.

We observed a small percentage of heterozygous SNPs and indels in the TH genome (1.37 % and 12.24 %, respectively). Possible explanations for the apparent presence of heterozygosity include errors in variant calling, true residual heterozygosity, and recent heterozygous mutations not yet fixed to homozygosity [46]. We did not consider heterozygous SNPs and indels in our subsequent analyses.

### Functional consequences of SNPs and indels

We assigned putative functional consequences to the set of homozygous variants (SNPs and indels) in TH using Variant Effect Predictor (VEP) [36], which classified the variants using terms from the Sequence Ontology (SO) [37] (Additional file 2: Table S2 and Additional file 3: Table S3). Variants which intersected multiple transcripts, and for which the set of SO terms associated with the variant differs among those transcripts, were categorized as “multiple classification sets”. For each of ten SO terms we designated “potentially pathogenic” (see Methods), we counted the number of variants in the “multiple classification sets” category that classified under that SO term in one or more transcripts.

**Fig. 2:**
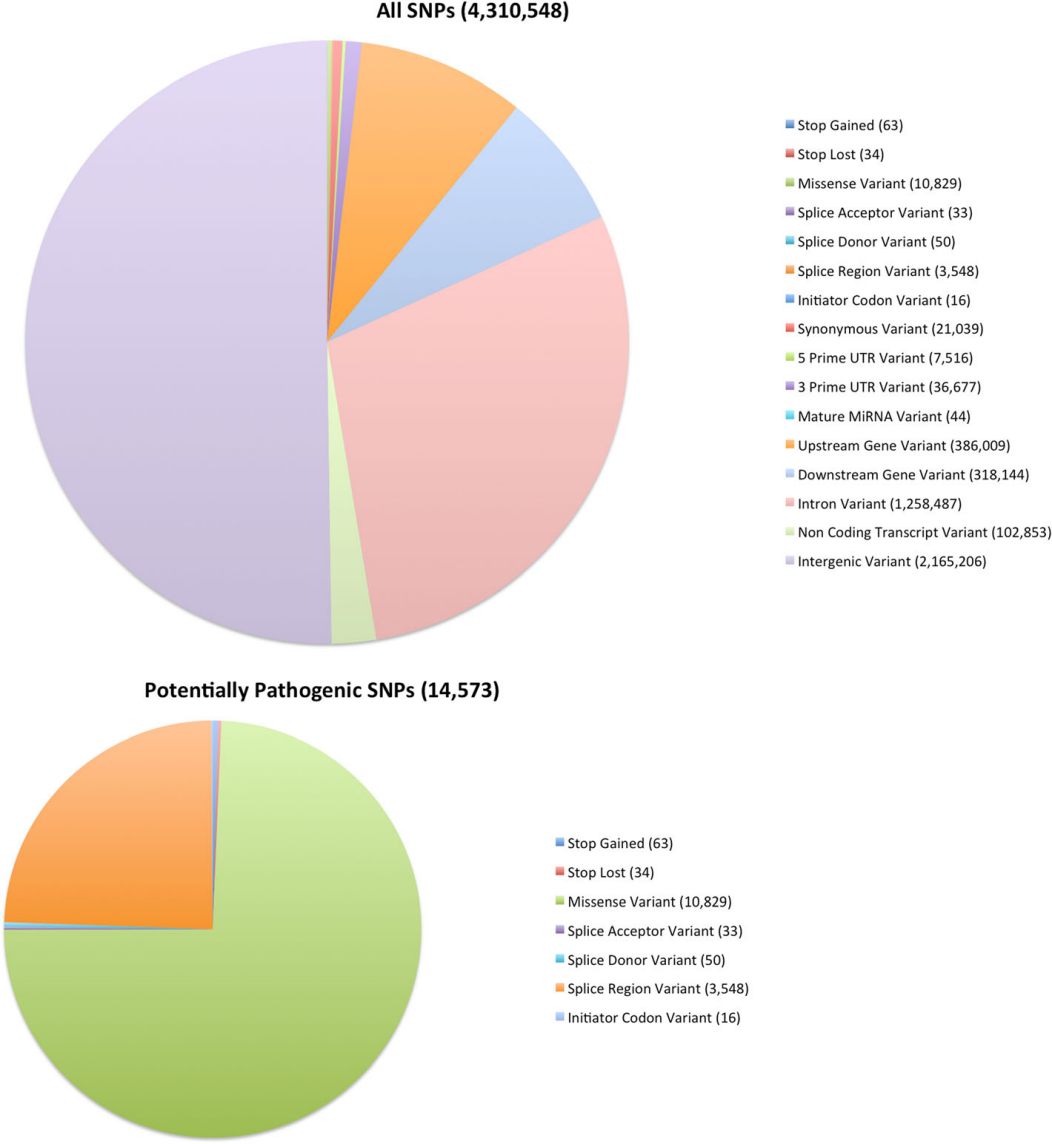
Classification of SNPs by Sequence Ontology term. The large pie chart shows the characterization of all TALLYHO/Jng SNPs relative to the C57BL/6 reference genome; the smaller chart shows the distribution of the subset of these we term “potentially pathogenic”, as described in the methods

**Fig. 3:**
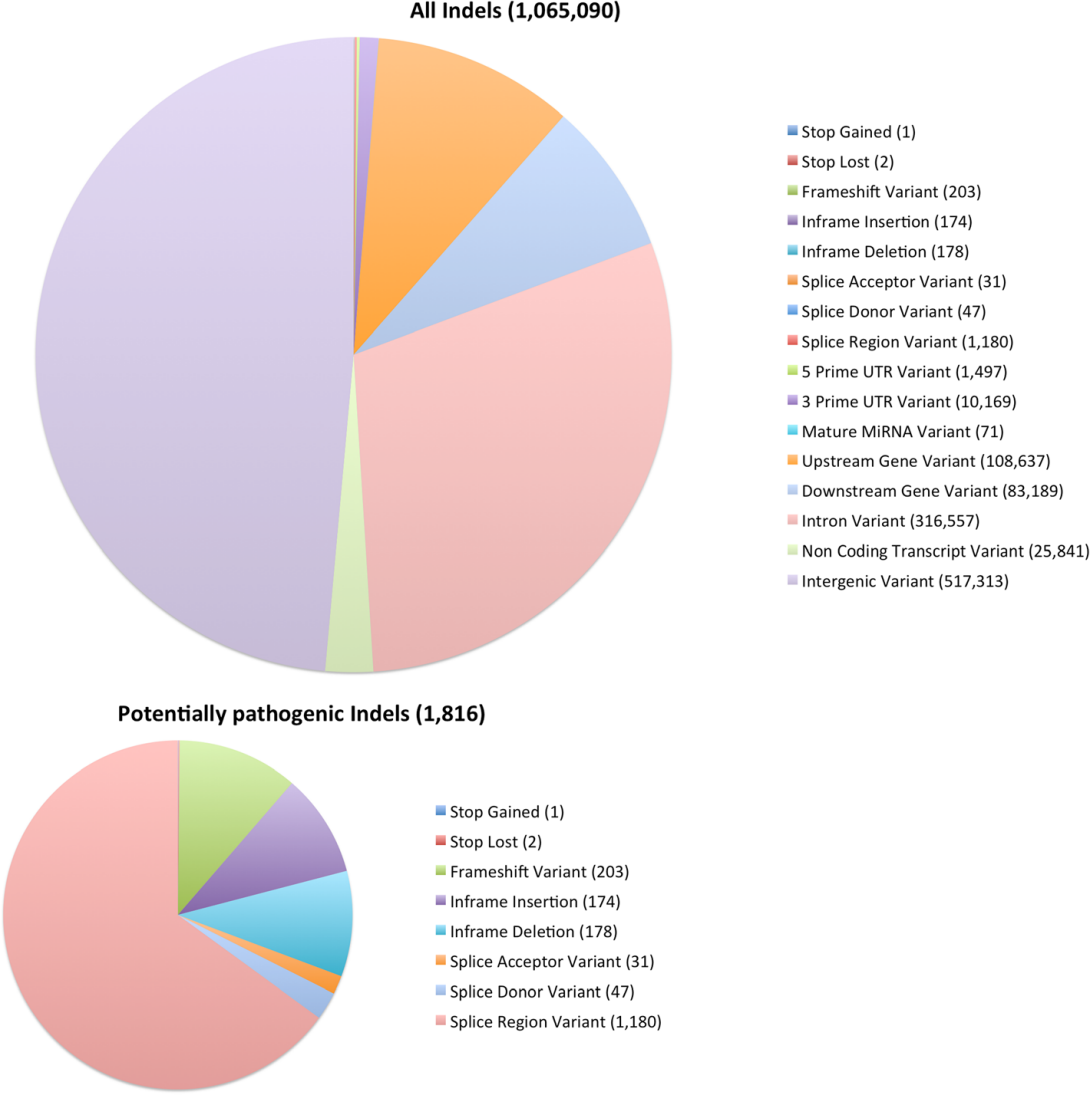
Classification of Indels by Sequence Ontology term. The large pie chart shows the characterization of all TALLYHO /Jng indels relative to the C57BL/6 reference genome; the smaller chart shows the distribution of the subset of these we term “potentially pathogenic”, as described in the methods

For each distinct collection of SO terms identified for a variant, we chose a representative term (as shown in Additional file 2: Table S2 and Additional file 3: Table S3). For variants with multiple classification sets this potentially resulted in multiple representative SO terms: for these variants with multiple classifications we categorized the variant as the “most pathogenic” according to the prioritization defined in the methods. This resulted in a unique SO term for each variant: the distributions of these SO terms among SNPs and indels are shown in Figs. 2 and 3, respectively. The majority of SNPs were either intergenic (50.2 %) or intronic (29.2 %). The next two largest SNP groups were those which were located 5 kb upstream (8.95 %) or downstream (7.38 %) from a coding gene. A small number of SNPs (less than 1 % of the total) were located within the protein coding regions of gene bodies. SNP variants resulted in 21,039 synonymous codon changes, 10,829 non-synonymous codon changes, 63 conversions of coding codons to stop codons and 34 conversions of stop codons to coding codons.

There were 10,829 SNPs causing non-synonymous changes to one or more protein-coding transcripts in 4,351 genes. We evaluated the effect of the amino acid substitutions resulting from the SNPs using SIFT [35, 47] (as implemented by VEP) and PROVEAN [38]. These algorithms classify the changes as either deleterious (SIFT score < 0.05, or PROVEAN score < -2.5) or tolerated/neutral (SIFT/PROVEAN). SIFT classified 14.8 % of the non-synonymous substitutions (1601 substitutions) in 1,148 genes as deleterious. PROVEAN classified 9.6 % (1041 substitutions) in 772 genes as deleterious. 4.8 % of the missense variants (512 substitutions) in 444 genes were classified as deleterious by both algorithms.

As expected, the vast majority (99.83 %) of TH indels fell into noncoding groups (either intergenic, intronic, 5 kb upstream or downstream of coding genes, 5′ or 3′ UTR, or non-coding transcript variants). The total in-frame and frameshift indels were 362 and 203, respectively (Fig. 3). Proportions of SNPs and indels in each functional class are similar to those found in the FVB/NJ genome [30] (Table 1).

**Table 1:** Comparison of distribution of variants in TALLYHO/Jng to that in FVB/NJ

	FVB/NJ		TALLYHO/Jng	
	Percentage of all SNPs	Percentage of all indels	Percentage of all SNPs	Percentage of all indels
5 kb upstream or downstream	7.731 %	9.895 %	7.496 %	8.167 %
5′ or 3′ UTR	0.434 %	0.433 %	0.291 %	0.298 %
Intronic	19.360 %	19.880 %	18.896 %	19.057 %
Synonymous coding	0.307 %		0.204 %	
Non-synonymous coding	0.176 %		0.103 %	
Essential splice site	0.000 %	0.000 %	0.000 %	0.001 %
Stop gain	0.002 %	0.000 %	0.000 %	0.000 %
Stop lost	0.000 %	0.000 %	0.000 %	0.000 %
In-frame codon insertion or deletion		0.016 %		0.011 %
Frameshift		0.014 %		0.005 %
Two or more consequences	15.436 %	15.710 %	20.401 %	21.467 %
Within, or 5Kb upstream or downstream from, non-coding gene or mature microRNA	6.240 %	6.280 %	2.362 %	2.403 %
Intergenic	50.314 %	47.772 %	50.246 %	48.591 %

### Comparison to the human genome-wide association study catalog and to known mendelian obesity genes

In order to interpret our data in the context of human disease, we collected human gene IDs in which variants were associated with obesity, diabetes, or metabolic syndrome, from the EBI GWAS catalog version 1.0.1 [39]. These gene IDs were mapped to orthologous mouse Ensembl gene IDs using the Ensembl interface to BioMart [40], generating sets of orthologous mouse genes. We additionally collected a list of known Mendelian obesity genes in humans from Pigeyre et al. [41], and generated a set of mouse orthologous genes as aforementioned. We then filtered the potentially pathogenic TH variants against these gene sets to provide lists of potentially pathogenic variants for each of these diseases. We found 26 genes with potentially pathogenic variants in the GWAS obesity gene set, 85 in the GWAS diabetes gene set, and 246 in the GWAS metabolic gene set. Additionally, we found 13 genes with potentially pathogenic variants orthologous to genes known to cause monogenic syndromic obesity, four orthologous to genes known to cause monogenic non-syndromic obesity, and three orthologous to genes known to cause monogenic non-syndromic lipodystrophy (Table 2, Additional file 5: Table S5).

**Table 2:** Variants in genes linked to traits of interest by GWAS or in Mendelian obesity genes

Source	Total genes	SNPs	Indels	Private SNPs	Private Indels
GWAS obesity	26	62	7	3	0
GWAS diabetes	85	208	27	7	2
GWAS metabolic syndrome	246	594	85	27	3
Monogenic syndromic obesity	13	45	2	0	0
Monogenic non-syndromic obesity	4	3	1	0	0
Monogenic non-syndromic lipodystrophy	3	4	0	0	0

### TH private SNPs and indels

We identified variants that were present in the TH strain and absent from 28 MGP strains by passing the bam files for each strain through our variant-calling pipeline at each location where we identified a TH variant. A TH SNP was identified as “private” if it was not called as the same SNP in any of the other 28 strains, and a TH indel was identified as “private” is no variant was identified at the same location in any of the other 28 strains. Additionally, the designation of “private” required a call quality at least 20 and read depth at least 5 in at least 21 of the 28 strains. In this analysis, we identified a total of 98,899 private SNPs and 163,720 private indels, which represented 2.29 % and 15.4 % of the total SNPs and indels, respectively.

**Fig. 4:**
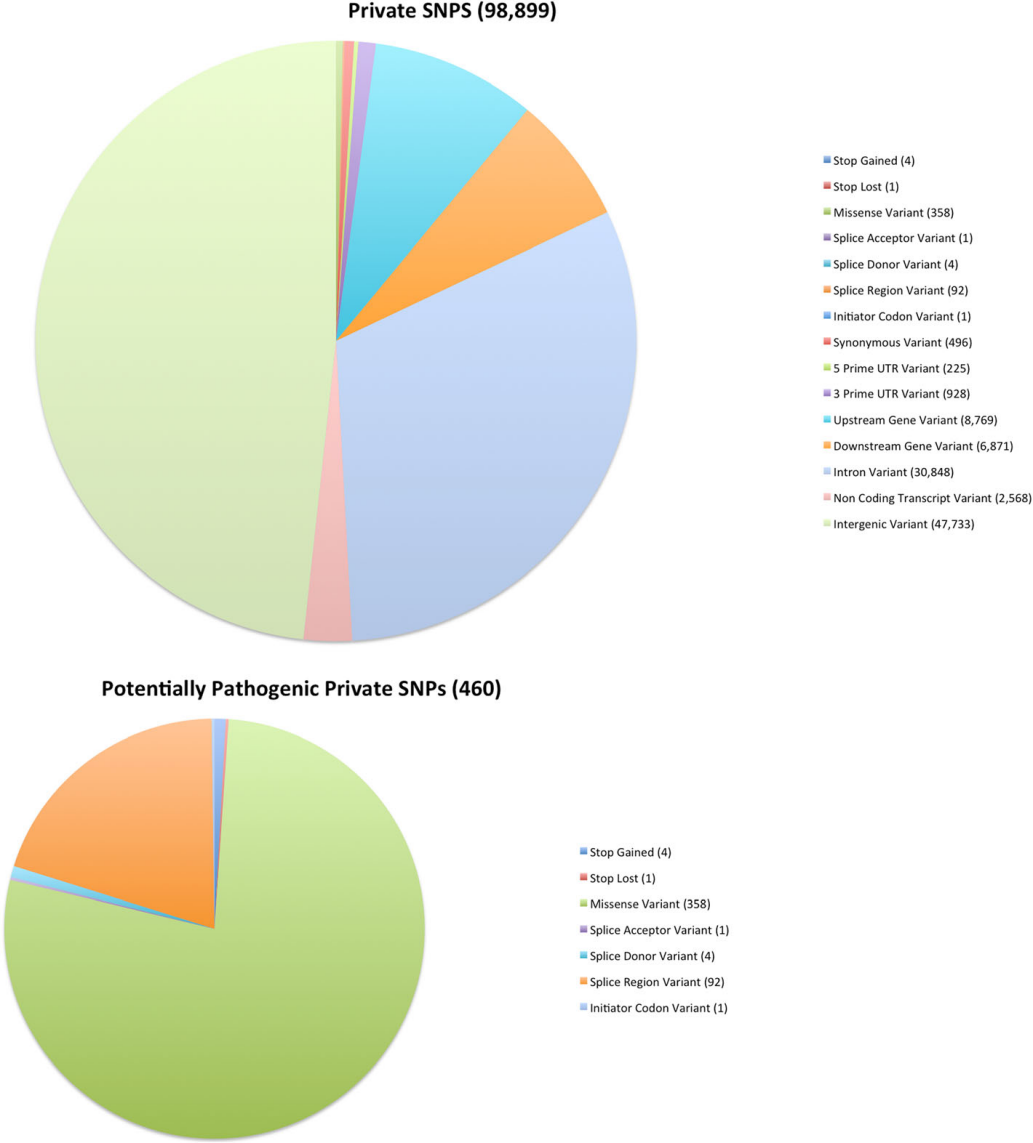
Classification of Private SNPs by Sequence Ontology term. The large pie chart shows the characterization of all TALLYHO/Jng SNPs relative to the C57BL/6 reference genome that are not present in 28 previously sequenced strains; the smaller chart shows the distribution of the subset of these we term “potentially pathogenic”, as described in the methods

**Fig. 5:**
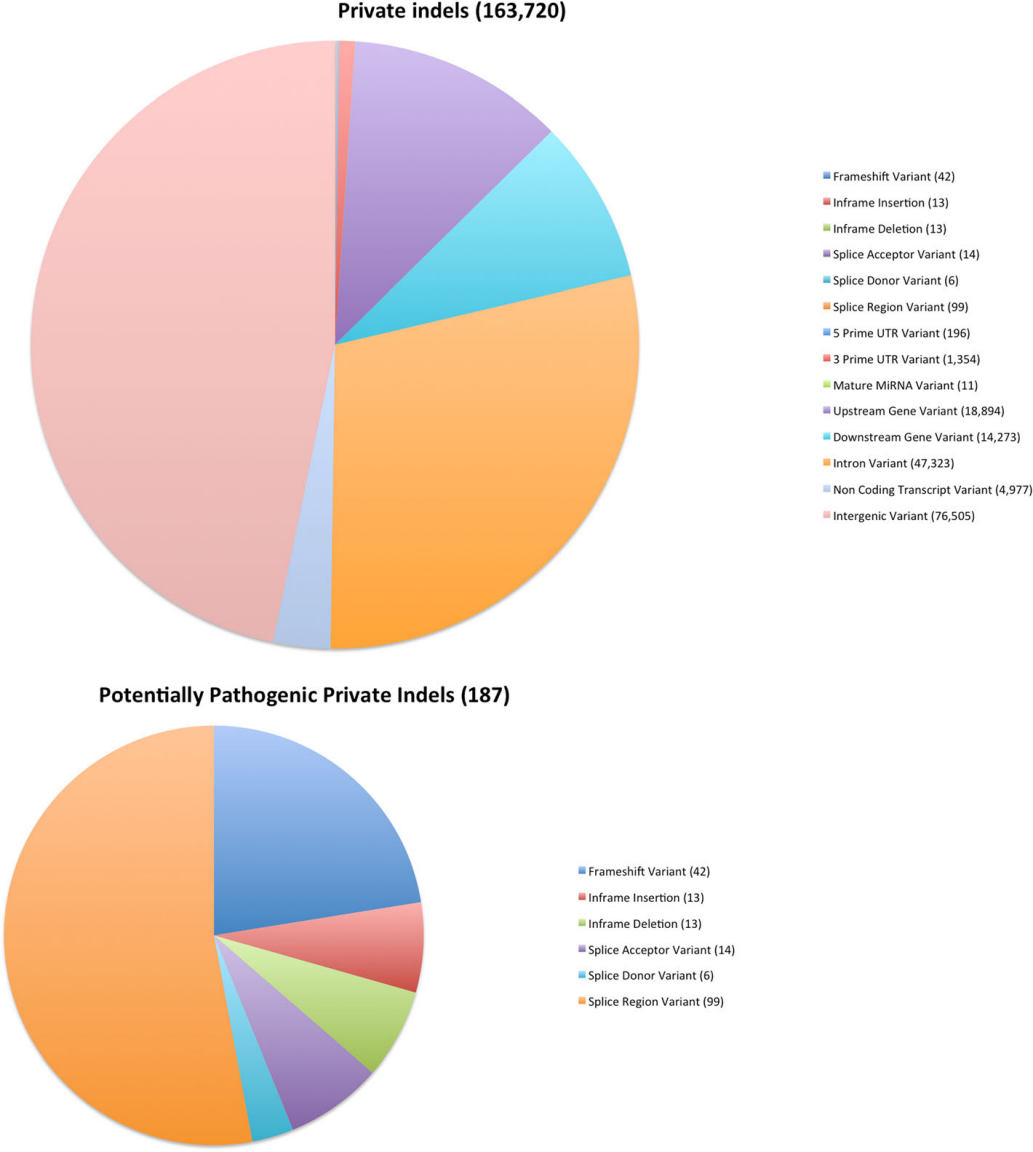
Classification of Private Indels by Sequence Ontology term. The large pie chart shows the characterization of all TALLYHO/Jng indels relative to the C57BL/6 reference genome that are not present in 28 previously sequenced strains; the smaller chart shows the distribution of the subset of these we term “potentially pathogenic”, as described in the methods

We classified private variants by SO term using VEP as described in the analysis for all variants above. Of the 98,899 private SNPs, 358 were missense substitutions, four resulted in the gain of a stop codon, and one in the loss of a stop codon (Additional file 6: Table S6 and Additional file 7: Table S7, Figs. 4 and 5). Among the 163,720 private indels, 42 were frameshift indels and 26 were in-frame indels (Additional file 6: Table S6 and Additional file 7: Table S7, Figs. 4 and 5). For these private SNPs and indels, along with those associated by VEP with splice regions and initiator codons, we counted the number of such variants occurring in each gene. There were 961 private SNPs meeting one or more of these criteria in 372 genes (Additional file 8: Table S8) and 576 private indels meeting one or more of these criteria in 215 genes (Additional file 9: Table S9). For the private SNPs, we found that 91 of the 372 genes had one or more private SNPs determined by SIFT to be deleterious missense variants, 69 had one or more private SNPs determined by PROVEAN to be deleterious, with 44 genes having SNPs determined to be deleterious by both algorithms.

In the analysis linking our variant lists with human GWAS studies and human Mendelian obesity genes described above, we determined the number of variants in orthologous genes which were private to the TH mouse (Table 2).

### Characterization of the *tabw2* obesity QTL interval on chromosome 6

*Tabw2* (TALLYHO associated body weight 2) is a major obesity QTL identified in TH mice, and confirmed by a congenic strain on the B6 background [48]. Using subcongenic analysis we have determined that the effect of *tabw2* on obesity could be attributed to two adjacent loci, *tabw2a* and *tabw2b* [49]. There are 411 and 73 protein coding genes cataloged in the Ensembl mouse annotation database in the *tabw2a* (6:80,217,217-125,356,646 in coordinates relative to GRCm38) and *tabw2b* (6:133,853,029-144,639,629) intervals, respectively. A total of 123,971 SNPs and 28,622 indels were found in the *tabw2a* and *tabw2b* intervals, of which 68,233 SNPs and 15,747 indels were in protein-coding genes (Table 3). Among those, only 5 SNPs (in 5 genes) in the *tabw2a* interval and 2 SNPs (in 2 genes) in the *tabw2b* interval were classified as deleterious by both SIFT and PROVEAN (shown in bold in Table 4). We then conducted a literature review of the biological function of the 29 genes in the *tabw2* interval with SNPs classified as deleterious by either algorithm, searching for potential link to obesity (Table 4). From this search, the *Cidec* gene, containing a SNP identified as deleterious by both SIFT and PROVEAN, drew our attention. CIDEC is a lipid droplet protein that is involved in the regulation of cellular lipid droplet size and lipid storage during lipid metabolism in adipocytes [50]. A loss of function mutation in CIDEC causes a familial partial lipodystrophy [43]. Further, weight loss via a low calorie diet was correlated with a reduced gene expression of CIDEC in adipose tissue in humans [51].

**Table 3:** Numbers of variants in the *tabw2* locus

	Region	
	*Tabw2a*	*Tabw2b*
Total SNPs	81,303	42,668
In protein coding genes	43,878	24,355
Potentially pathogenic	247	107
SIFT deleterious	22	8
PROVEAN deleterious	9	9
Total indels	20,814	7,808
In protein coding genes	11,302	4,445
Potentially pathogenic	38	11

**Table 4:** SNPs in the *tabw2* locus identified as deleterious by SIFT or PROVEAN

Gene	Location on Chr 6 (bp)	Codon Ref/TH	A.A. Ref/TH	Exon	SIFT score	PROVEAN score	Function	Ref
*Gcfc2*	81923813	tCt/tTt	S/F	1/18	0.05	-2.5	Unknown	[59]
	81933786	cCa/cAa	P/Q	5/18	0.02	1.05		
	**81941369**	**Cgg/Tgg**	**R/W**	**7/17**	**0**	**-5.41**		
*Ccdc142*	83107557	cCt/cTt	P/L	7/11	0.15	-2.97	Unknown	
*Htra2*	83053837	Ggg/Cgg	G/R	1/7	0	N/A	Serine protease	[60]
	83054296	cCt/cTt	P/L	1/7	0	-2.04		
*Mogs*	83118580	Cgc/Tgc	R/C	4/4	0.03	0.94	Endoplasmic reticulum glucosidase	[61]
*Rtkn*	831151299	Ccc/Gcc	P/A	12/13	0.14	-4.41	Rho effector protein	[62]
	83151312	gCc/gTc	A/V	11/12	0.03	-1.1		
*1700003E16Rik*	83162665	aCc/aTc	T/I	4/4	0.01	-1.12	Unknown	
*Tet3*	83404686	Ctt/Ttt	L/F	3/3	0.01	-1.65	5-methylcytosine oxidase	[63]
*Aak1*	86965533	aAt/aGt	N/S	14/21	0.09	-2.66	Endocytosis	[64]
*Gfpt1*	87053776	aCa/aTa	T/I	4/5	0	-0.48	Hexosamine biosynthesis	[65]
*D6Ertd527e*	87111649	ACc/CTc	T/L	2/2	N/A	-4.67	Unknown	
*Chchd6*	**89467434**	**Cgt/Tgt**	**R/C**	**5/8**	**0.03**	**-3.51**	Mitochondrial protein	[66]
*Zfyve20*	92199851	cGg/cAg	R/Q	7/12	0.02	-1.32	Regulate the intracellular route of internalized receptors	[67]
*Lmod3*	97252294	aGt/aAt	S/N	1/3	0.02	-0.55	Actin thin filament length regulation	[68]
*Ttll3*	113409684	Ggg/Agg	G/R	2/2	0.04	-1.33	Glycylases	[69]
*Cidec*	**113433131**	**Cgt/Agt**	**R/S**	**3/6**	**0**	**-5.85**	Lipid storage and lipid droplet formation	[70]
*Irak2*	113647785	Ctg/Gtg	L/V	2/12	0	-2.4	Toll-like receptor signaling	[71]
*Rpl28-ps4*	117213939	cAa/cGa	Q/R	1/1	0	-0.09	Unknown	
*Ret*	**118179286**	**aCc/aTc**	**T/I**	**5/20**	**0**	**-3.05**	Proto-oncogene	[72]
*Cacna1c*	118602267	aCt/aAt	T/N	42/47	0.43	-2.93	L-type Ca^2+^ channel	[73]
*Cecr5*	120510398	Cgt/Tgt	R/C	8/8	0.04	-1.85	Unknown	
*A2m*	121639361	atG/atA	M/I	3/36	0.02	-0.6	Protease	[74]
	**121661523**	**aCg/aAg**	**T/K**	**21/36**	**0**	**-5.88**		
*8430419L09Rik*	135209106	tCg/tTg	S/L	2/2	N/A	-6.0	Unknown	
	135209130	aTg/aCg	M/T	2/2	N/A	-6.0		
	135209172	cAg/cGg	Q/R	2/2	N/A	-4.0		
	135209250	cGg/cAg	R/Q	2/2	N/A	-4.0		
	135209265	gCc/gTc	A/V	2/2	N/A	-4.0		
*Atf7ip*	136561386	tCg/tTg	S/L	3/4	0.01	-0.72	Transcriptional cofactor	[75]
	136561422	aGt/aCt	S/T	3/4	0.05	-0.12		
*BC049715*	136840037	Act/Cct	T/P	1/1	0.11	-3.22	Unknown	
	136840106	Aga/Gga	R/G	1/1	0.37	-2.86		
	**136840191**	**aTg/aCg**	**M/T**	**1/1**	**0.01**	**-4.56**		
*Art4*	136857180	Ccc/Acc	P/T	1/3	0.02	-1.15	ADP-ribosyltransferase	[76]
*Mgp*	136872651	aAa/aGa	K/R	4/4	0.02	-0.33	Calcification inhibitor	[77]
*Ptpro*	137368083	aCg/aTg	T/M	2/27	0.03	-1.93	Tumor suppressor	[78]
*Igbp1b*	138658041	aCg/aTg	T/M	1/1	0.05	-1.27	Regulation of phosphatase activity in neuronal cells	[79]
*Pik3c2g*	**139967890**	**Agt/Tgt**	**S/C**	**16/16**	**0.01**	**-2.86**	Long-term Akt2 activation	[80]

*Chr* chromosome, *A.A.* amino acid, *Ref* reference, *TH* TALLYHO

SNPs predicted to be deleterious by both SIFT and PROVEAN are shown in bold

**Fig. 6:**
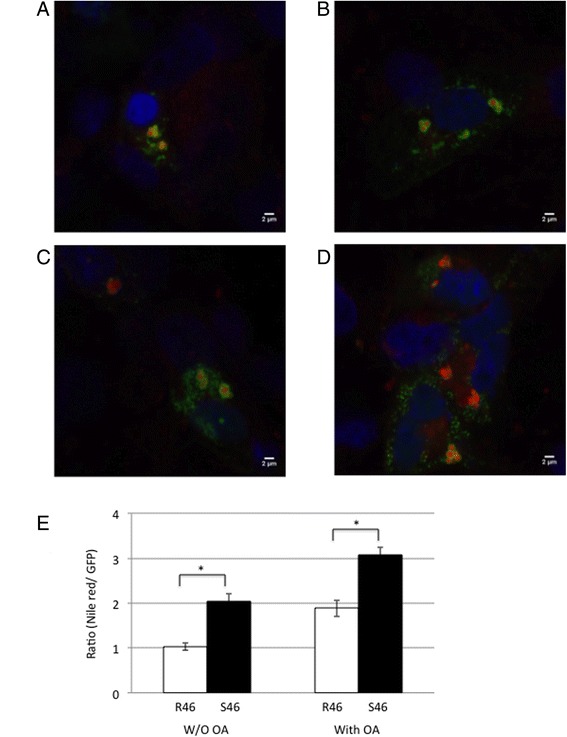
Representative images of lipid droplets in COS-1 cells transfected with (**a** and **b**) AcGFP1-CIDEC (R46) and (**c** and **d**) AcGFP1-CIDEC (S46). **a** and **c** present cells cultured in media without oleic acid (OA) and **b** and **d** present cells cultured in media with OA. Images of nuclei labeled with DAPI, neutral lipid labeled with Nile red and location of AcGFP1 were merged. **e** Quantification of lipid specifically associated with GFP in cells. Using ImageJ software, the ratio of Nile red/GFP signal was calculated from 34 lipid droplet clusters each for R46 and S46 without OA and 47 lipid droplet clusters for R46 and 46 lipid droplet clusters for S46 with OA. Data are presented as means ± SEM. *P < 0.05

There was one nucleotide substitution in the *Cidec* coding sequence, 136 C > A. The substitution of 136 C > A resulted in an amino acid difference of R46S (Arginine 46 Serine) between B6 and TH strains (R46 in B6; S46 in TH). To examine the function of the *Cidec* R46S polymorphism, we created murine plasmids AcGFP1-CIDEC (R46) and AcGFP1-CIDEC (S46) and transiently transfected them into COS-1 cells. COS-1 cells have been used as models for monitoring CIDEC function in prior studies because they lack endogenous Cidec activity [42–44]. It was apparent that AcGFP1-CIDEC was localized to distinct lipid droplets stained with Nile red (Fig. 6). When we considered Nile red lipid staining specifically associated with GFP fluorescence (co-localization), COS-1 cells transfected with the S46 variant exhibited an increase in lipid accumulation compared to cells transfected with the wild type R46 variant in the media both with and without oleic acid (Fig. 6). We speculate that the S46 variant may be hypermorphic in that it enhances the function of CIDEC in promoting lipid accumulation in lipid droplets.

## Discussion

### Landscape of the TALLYHO genome

We found a total of 5,375,638 variants (SNPs and indels) in the TH genome, compared to the reference B6 genome (GRCm38). As expected, the large majority of variants occurred in non-protein coding regions of the genome. The number of SNPs was broadly consistent with that reported by Keane et al. and Wong et al. for 13 laboratory strains [30, 34], and substantially fewer than the four wild-derived strains (PWK/PhJ, CAST/EiJ, WSB/EiJ, and SPRET/EiJ) sequenced in the Keane et al. study [34]. We found 3.5 % to 33.6 % more indels when compared to those 13 laboratory strains, and again considerably fewer than the four wild-derived strains. The proportion of private SNPs we observed was higher than 10 of the laboratory strains from Keane et al. [34], with the two exceptions being the two strains NOD/ShiLtJ and NZO/HILtJ. We also showed a lower proportion of private SNPs in the TH strain than discovered in FVB/NJ [30]. The proportion of private indels we observed in TH was higher than in these studies. Although the origins of the Theiler Original mouse are unknown, the generally higher proportion of private variants in TH (compared to most laboratory strains) is consistent with the possibility that TH has a unique ancestor. We also note that our sequencing coverage of 64.8X was higher than in these other studies, increasing our general power to detect variants.

### Classification of variants

We provided a comprehensive catalog of variants of the TH mouse, relative to the B6 mouse, classified according to the effect of the variant on protein sequence. These effects were predicted by comparing the location of the variant on the reference genome to known genomic annotations provided by the Ensembl gene database [52]. While it is relatively straightforward to automate the classification of a large number of variants via genomic annotations on a coarse scale, providing detailed interpretations of the effect of some mutations requires greater inspection of the context of the mutation that cannot be readily automated and performed on a genome-wide scale. For example, the effect of intronic mutations is not well characterized or understood at present. Our aims in this study include both providing a general overview of the landscape of the TH mouse genome relative to the reference genome, and also providing sufficient detail of the nature of the 5.3 million variants discovered. To this end, we used an automated process to classify variants into a large number of precisely defined categories, and then subsequently reduced the number of these categories, subjectively categorizing them into a smaller set of representative terms. The first categorization provides a fine-grained resource for searching for individual variants that may have phenotypic consequences, and the second provides a general overview of the landscape of the TH genome.

The Sequence Ontology (SO) [37] provides a controlled vocabulary for describing features and annotations associated with a biological sequence, including mutations at the sequence level, along with a formalized description of the relationships between these terms. The Variant Effect Predictor (VEP) [36] is a tool that predicts the effect of genomic variants by analyzing the variant in the context of annotations on the reference genomic sequence. For each variant and each known transcript containing that variant, VEP outputs a subset of SO terms associated with that variant. In many cases, this results in multiple SO terms being associated with a single variant in the context of a single transcript. For example, we found an A to G single nucleotide polymorphism (SNP) at genomic location 126,585,377 on chromosome 7. This SNP occurs in the second nucleotide of the first codon of the second exon of the apolipoprotein B receptor gene, *Apobr*, mutating the GAC codon to GGC. Since this mutation results in a change in amino acid from aspartic acid to glycine, the SNP is annotated with the SO term “missense_variant”. Since this variant also occurs within three bases of a splice site, it is also annotated with the SO term “splice_region_variant”. In order to provide a complete, unbiased characterization of the types of variants discovered, we list all unique combinations of SO terms for a single variant and the number of times those combinations occur (Additional file 2: Table S2 and Additional file 3: Table S3).

In order to provide a concise overview of the relative proportions of occurrences of the major types of variants, for each unique combination of SO terms we selected a “representative” SO term (Additional file 2: Table S2 and Additional file 3: Table S3, second column). These overviews are presented in Figs. 2 and 3.

Due to the presence of multiple transcripts for some genes, and due to the presence of overlapping genes, some individual variants are present in multiple transcripts. In this case, VEP outputs a potentially different combination of SO terms for each of the transcripts in which the variant occurs. When there are multiple, distinct, combinations for a given variant due to its presence in multiple transcripts, these variants are categorized in Additional file 2: Table S2 and Additional file 3: Table S3 as “multiple classification sets”. In order to uniquely classify such variants in the overview (Figs. 2 and 3), we picked a representative SO term for each set of SO terms using the strategy shown in Additional file 2: Table S2 and Additional file 3: Table S3, and then chose the most pathogenic out of these representative SO terms using the priority described in the methods.

### Filtering variants by the human GWAS catalog

We employed a translational methodology to identify and prioritize candidate genes for complex disorders for which TH is a model. We examined all the TH variants for potential link to relevant human disease using the EBI GWAS catalog [39] and generated enriched candidate gene lists for obesity, diabetes and metabolic syndrome. We reviewed biological function for selected genes and identify genes we could connect to obesity and/or diabetes. GLIS3 is a transcription factor that plays an important role in pancreatic development and insulin gene expression in beta cells [53]. SORBS1 is involved in insulin stimulated glucose transport in adipocytes [54]. IGF2BP2 is a RNA binding protein, participating in posttranscriptional RNA processing, i.e., RNA splicing, stabilization, transport, and translation [55]. Inactivation of the *Igf2bp2* gene caused a resistance to diet-induced obesity in mice [56]. Semaphorin 5A (*Sema5A*) is an axon regulator molecule and plays a role during neuronal and vascular development [57]. Collectively, our convergent approaches systematically integrate whole genome sequencing data and genetic information from GWAS-derived findings and provide an opportunity to discover candidate genes for further functional validation.

### Missense polymorphism of R46S in CIDEC between B6 and TH mice

Among the genes with SNPs that were identified as deleterious by both SIFT and PROVEAN (Additional file 5: Table S5), were orthologous to Mendelian lipodystrophy genes, and were contained within the *tabw2* interval, we identified *Cidec*, which is an important regulator of energy homeostasis directly involved in promoting the accumulation of triglyceride into intracellular lipid droplets [50]. Lipid droplets are spherical organelles found in many types of eukaryotic cells, including adipocytes, and are composed of a core of neutral lipids, such as sterol esters or triglycerides, surrounded by a monolayer of phospholipids, free cholesterol, and multiple specific proteins including CIDEC [50]. In adipocytes, cellular energy is stored as triglycerides in lipid droplets, and in conditions of fatty acid excess, lipid droplets rapidly increase their volumes [58]. The capacity of CIDEC in accumulating triglycerides into intracellular lipid droplets was demonstrated by transfection experiments using multiple cell types including C pre-adipocytes, 293T cells, and COS cells [42–44]. For example, transfection of full-length murine *Cidec* into COS-7 cells increased total cellular triglycerides by 50 % [44]. We found that the TH allele of CIDEC S46 variant allowed for more lipid accumulation than the B6 allele of CIDEC R46 variant in COS-1 cells. Further functional validation of this missense polymorphism in vivo may provide an opportunity to understand the role of *Cidec* in the context of obesity.

## Conclusions

We have sequenced the whole genome of obese type 2 diabetic TH mice by next-generation sequencing and generated a complete catalog of variants classified by location relative to genes and predicted consequences for protein products. We filtered the list of variants to the *tabw2* obesity QTL and identified a missense polymorphism in *Cidec* whose protein function we characterized. Furthermore, by comparing the variant catalog to the human GWAS catalog and to known human Mendelian obesity genes, we were able to identify a list of susceptibility candidate genes that could be used to dissect the components of polygenic diseases such as obesity and type 2 diabetes.

## Additional files

Additional file 1: Table S1. Mouse genome project strains. List of strains from the Mouse Genome Project used in the determination of “private” variants. (DOCX 43 kb)
Additional file 2: Table S2. Classification of TH SNPs by sets of SO terms associated with the SNP. (XLSX 9 kb)
Additional file 3: Table S3. Classification of TH indels by sets of SO terms associated with the indel. (XLSX 10 kb)
Additional file 4: Table S4. PCR primer sequences used for validation of selected variants. (XLSX 56 kb)
Additional file 5: Table S5. Variants in orthologs of GWAS-associated and Mendelian Genes. List of orthologous genes to human genes associated by GWAS studies to obesity, diabetes, and metabolic syndrome, and to Mendelian obesity genes, with variant type and number. (XLSX 74 kb)
Additional file 6: Table S6. Private SNP classification. Classification of private TH SNPs by sets of SO terms associated with the SNP. (XLSX 8 kb)
Additional file 7: Table S7. Private indel classification. Classification of private TH indels by sets of SO terms associated with the indel. (XLSX 8 kb)
Additional file 8: Table S8. Genes with pathogenic private SNPs. List of genes with pathogenic private SNPs, with SIFT scores and SO terms for the corresponding SNP. (XLSX 64 kb)
Additional file 9: Table S9. Genes with pathogenic private indels. List of genes with pathogenic private indels, with SO terms for the corresponding indel. (XLSX 54 kb)

## Abbreviations

DNA: Deoxyribonucleic acid
EBI: European Bioinformatics Institute
GWAS: Genome Wide Association Study
Indel: Insertion and/or Deletion
Mbase: Megabase (1,000,000 bases)
MGP: Mouse Genome Project
NCBI: National Center for Biotechnology Information
PROVEAN: Protein Variation Effect Analyzer
QTL: Quantitative Trait Locus
SIFT: Sorting Intolerant From Tolerant
SNP: Single Nucleotide Polymorphism
SO: Sequence Ontology
SRA: Sequence Read Archive
Tabw2: Tallyho Associated Body Weight 2
TH: TALLYHO/Jng mouse strain
TJL: The Jackson Laboratory
VEP: Variant Effect Predictor
