# Supplementary material for: Whole genome sequence analysis of the TALLYHO/Jng mouse
Source: BMC Genomics. 2016 Nov 11;17:907. doi: 10.1186/s12864-016-3245-6 (PMC5106808; doi:10.1186/s12864-016-3245-6)
Supplement: Additional file 1: Table S1. — Mouse genome project strains. List of strains from the Mouse Genome Project used in the determination of “private” variants. (DOCX 43 kb) [file 12864_2016_3245_MOESM1_ESM.docx]

Supplementary Table S1. Mouse strains in the Mouse Genome Project used for identification of private variants.

| Strain |
| --- |
| 129P2_OlaHsd |
| 129S1_SvImJ |
| 129S5SvEvBrd |
| A_J |
| AKR_J |
| BALB_cJ |
| BUB_BnJ |
| C3H_HeJ |
| C57BL_10J |
| C57BL_6NJ |
| C57BR_cdJ |
| C58_J |
| CAST_EiJ |
| CBA_J |
| DBA_1J |
| DBA_2J |
| FVB_NJ |
| I_LnJ |
| LP_J |
| MOLF_EiJ |
| NOD_ShiLtJ |
| NZB_B1NJ |
| NZO_HlLtJ |
| NZW_LacJ |
| PWK_PhJ |
| SEA_GnJ |
| SPRET_EiJ |
| WSB_EiJ |
